# The paradox of food production, expenditure, poverty and stunting  in Tanzania: an ecological study design

**DOI:** 10.12688/f1000research.74295.3

**Published:** 2026-02-26

**Authors:** Novatus Tesha, Malale Tungu, Alphoncina Kagaigai, Boniface Yohana, Hevenlight A. Paulo

**Affiliations:** 1 Development of Development Studies, Muhimbilili University of Health and Allied Sciences, Dar es Salaam, Tanzania; 2 Department of National Accounts, National Bureau of Statistics, Dodoma, Tanzania; 3 Department of Biostatistics and Epidemiology, Muhimbili University of Health and Allied Sciences, Dar es Salaam, Tanzania

**Keywords:** Stunting, Food Production, Basic Need Poverty and Household Consumption

## Abstract

**Background:**

There have been claims amongst nutrition stakeholders in Tanzania that the food basket regions, are the regions most affected by stunting among children. However, this study could not find evidence that combines food production and stunting levels, to substantiate this claim. Therefore, this study aims to compare data on stunting, food production and consumption within administrative regions of the Tanzania mainland.

**Methods:**

The study used an ecological study design to show the relationship between stunting, poverty, food production and food and non-food consumption expenditure across administrative regions in Tanzania mainland. The study used data from three national wide surveys: 2017/2018 Household Budget Survey (HBS), Tanzania National Nutrition Survey (TNNS) 2018 and Agriculture Statistics for Food Security report 2018/2019.

**Results:**

The study showed that there is a positive relationship between the prevalence of stunting and food production (r=0.43, p=0.03), while there is a negative relationship between stunting and the level of both the average monthly household food consumption expenditure (r = -0.51, p = 0.01) and average monthly household non-food consumption expenditure (r = -0.51, p = 0.01). Furthermore, some regions with high stunting prevalence (e.g., Njombe) were found to have relatively low basic-needs poverty rates.

**Conclusion:**

The study found a positive relationship between food production and the prevalence of stunting using data across regions in mainland Tanzania. This is an indication that regional food production may not entail nutrition outcomes, hence a call for more advocacy on nutrition-sensitive agriculture.

## Introduction

Stunting, defined as being too short for one’s age, is among the important indicators to track children’s malnutrition. ^1^ Globally, the number of stunted children is decreasing, however, its prevalence level is still unacceptable. In the past 18 years, the number of stunted children globally, decreased by 49.2 million. It has been reported that in 2018, over 149 million (21.9%) children were estimated to have stunted growth globally, compared to 198.2 million children in 2000. ^2^ Most of the stunted children live in developing countries, especially in Asia and Africa with 55% and 39% of children suffering from stunting, respectively. Africa is the only region where the number of cases is increasing; rising from 50.3 million in 2000 to 58.8 million in 2018 with disparities existing at the sub-national level. ^2^^,^^3^

The prevalence of stunting in Tanzania decreased by 18% from the 1990s, from 50% of children to 32% in 2018. ^4^ This rapid change was due to the different strategies taken by the government, in collaboration with development partners, including implementation of the National Nutrition Strategy 2011/2012 to 2015/2016 ^5^ and the National Multi-Sectoral Nutrition Action Plan 2016 – 2021. ^6^ However, this level is still high as per the World Health Organization (WHO) recommended thresholds (>= 30%). WHO defines levels of stunting in four levels: low (<20%); Medium (20 – 29%), High (30 – 39%) and very high (>=40). ^13^^,^^14^ Despite the decrease in the prevalence level, the number of stunted children is increasing. It increased from 2.5 million in 2005 to 3 million in 2018. ^7^ The disparity exists across regions in Tanzania Mainland. Out of 26 regions, 15 regions have a stunting prevalence of above 30%, among them, six regions have above 40%. The regions with the highest stunting levels are Njombe (53%), Rukwa (47.9%), Iringa (47.1%), Songwe (43.3), Kigoma (42.2) and Ruvuma 41%. ^8^

The reviewed literature has shown that under-five stunting can be explained by different immediate and intermediate factors. Some of the factors include income levels, family size, education level of the mother, sex of the baby, age of the baby, feeding practices, food preparations, overall health status, types of occupation of the mother, weight of the baby and age of the mother at birth. ^9^^–^^12^ The underlying factors of stunting are government policies, pricing of food, access to social services, farming practices and economic status of the household. ^6^ Household poverty level and other factors related to poverty are also cited as determinates of stunting at household levels. ^11^^,^^12^^,^^14^

In Tanzania, some regions that are leading in food production and with a small prevalence of poverty, are also leading in the prevalence of stunting. For example, Njombe has the highest level of stunting (53%) and yet, it is the region with the third lowest rate of poverty (13.2%), just above are Dar es Salaam and Kilimanjaro with 8% and 10% levels respectively. ^4^^,^^15^ Similarly, in 2018, Ruvuma was the highest region in terms of food production but it had a stunning level of 41%. There has been a debate amongst nutrition stakeholders (those working in the government, United Nations Agencies and donor countries supporting nutrition interventions, academia and non-governmental organization in nutrition), on the controversy on regions with both high food production and stunting levels. However, to the best of our knowledge, we have not found previous research conducted to investigate this. Therefore, this study aims to compare data on stunting, food production and consumption across administrative regions of Tanzania.

## Methods

### Study design and data source

This is an ecological study design using secondary data compiled from three sources: Tanzania Household Budget Survey (HBS) 2017/2018, ^15^ Tanzania National Nutrition Survey (TNNS) 2018 ^4^ and Agriculture Statistics for Food Security report 2018/2019. ^16^ Both HBS and TNNS are national-wise cross-sectional data organized by the Ministry of Finance and Planning and Ministry of Health respectively, while Agriculture Statistics for Food Security report 2018/2019 was prepared by the Ministry of Agriculture.

### Study setting

**Figure 1.**
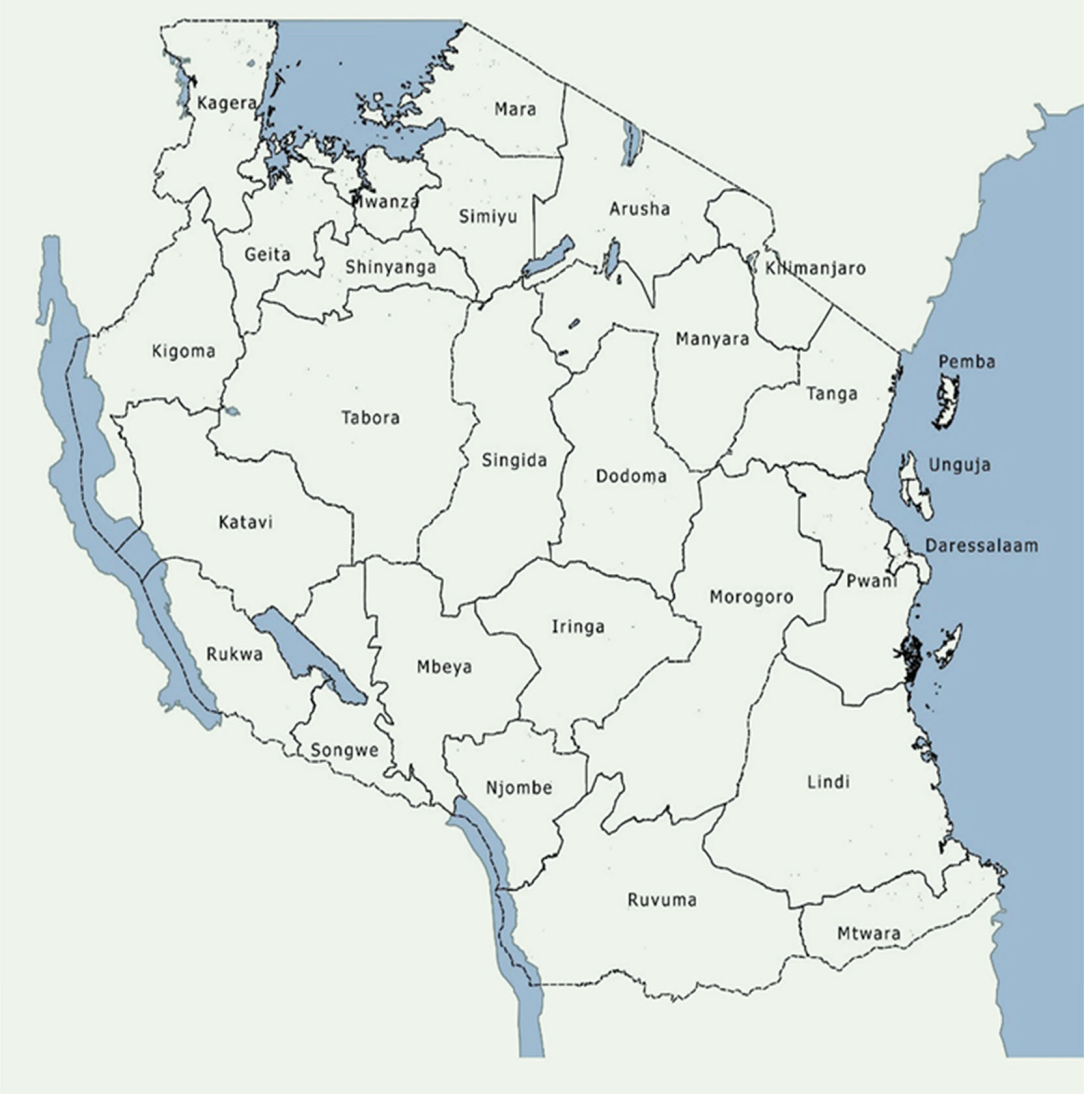
The map of Tanzania with the administrative regions.

Data for this study was sourced from 26 Tanzania mainland administrative regions. The regions were chosen because currently, aggregated data are representative at the regional level. Similarly, regions were chosen to determine whether food production could be a determinant of stunting at the regional level and hence answering the paradox ( Figure 1).

### A sampling of the Household Budget Survey and Tanzania National Nutrition Survey

The 2017/18 HBS had a sample of 9,552 individuals, which allowed disintegration of the results at the regional level. The data set adopted a two-stage cluster sample design. The first stage involved the selection of enumeration areas, Primary Sampling Units, (PSUs) from the 2012 Population and Housing Census Frame. ^15^ The second stage involved systematic sampling of households from the updated PSUs list. All household members, regardless of their age, who were the usual members of the selected households and all the visitors who were present in the household on the night before the survey, were eligible. The survey took place from 1 ^st^ December 2017 to 30 ^th^ November 2018.

TNNS used two-stage cluster sampling using Probability Proportional to Size (PPS), a representative at the regional and national level. The first stage sample of clusters was drawn independently for each domain. The second stage of sampling consisted of selecting households within each selected cluster, by using a systematic random selection procedure. Data were collected from the sample of 17,524 children aged 0-59 months and 9,426 women aged 15-49 years old. Data collection took place from 25 ^th^ of September and finished on the 17 ^th^ of November 2018.

The agriculture statistics were obtained from the agricultural statistics for food security report. This is the national wide report which synthesis data collected through various ways including 10 different tools. Among the tools were a tool for target and implementation of crop cultivation at field level, food availability at local markets and food security questinnaire 1 (FSQ1). ^16^ The foods which were included in this reports include cereal and non-cereal. For cereal food it included maize, sorghum, finger millet, bulrush millet, rice, wheat and cereals. The non-cereals include beans, peagon peas, cowpeas, groundnuts, greengram, bambaranuts, cassava, banana, sweet potatoes and round patatoes.

### Variables

The outcome variable of this study is the regional prevalence of stunting. Children were reported to be stunted if their height-for-age is more than two standard deviations below the World Health Organization’s Child Growth Standards median. ^16^ The exposure variables were the regional food production (cereal and non-cereals food crops in metric tons), the proportion of basic need poverty, and average monthly household food and non-food consumption expenditure.

### Data management and analysis

Data were cleaned and analyzed using Microsoft Excel and STATA version 15. Mean and standard divisions were used to summarize the numerical variables. Correlation coefficients and scatter plots were used to determine the linear relationship between outcome (regional prevalence of stunting) and exposure variables (food production, average household monthly food and non-food consumption expenditure and proportion of regional basic need poverty). Findings were presented using tables, figures and narrations. The significance level was set at 5%.

## Results

### Summary statistics of the variables

The overall prevalence of stunting for the Tanzania mainland is 33.5%, ranging from 20% in Kilimanjaro to 53.6% in the Njombe region. Basic need poverty stands at 27.5%, ranging from 8% in Dar es Salaam to 45% in the Rukwa region. On the other hand, the average food production is 977,544.80 Metric tons per year and the mean average monthly household food consumption expenditure is 47,986 ranging from 34,354 in Kigoma to 70,966 in Dar es Salaam (see Table 1).

**Table 1.** Summary statistics of the variables (n = 26).

Variable	Mean/prevalence	Standard deviation
Stunting (overall prevalence)	33.5	8.8
Food production (metric tons)	977,544.8	434,201.7
Basic need poverty	27.6	9.0
Average monthly household food consumption expenditure (TZS)	47,986 (USD 20.8)	8,603 (USD 3.7)
Average monthly household food consumption expenditure	41,345 (USD 17.9)	8,603 (USD 3.7)

### Stunting and food production

**Figure 2.**
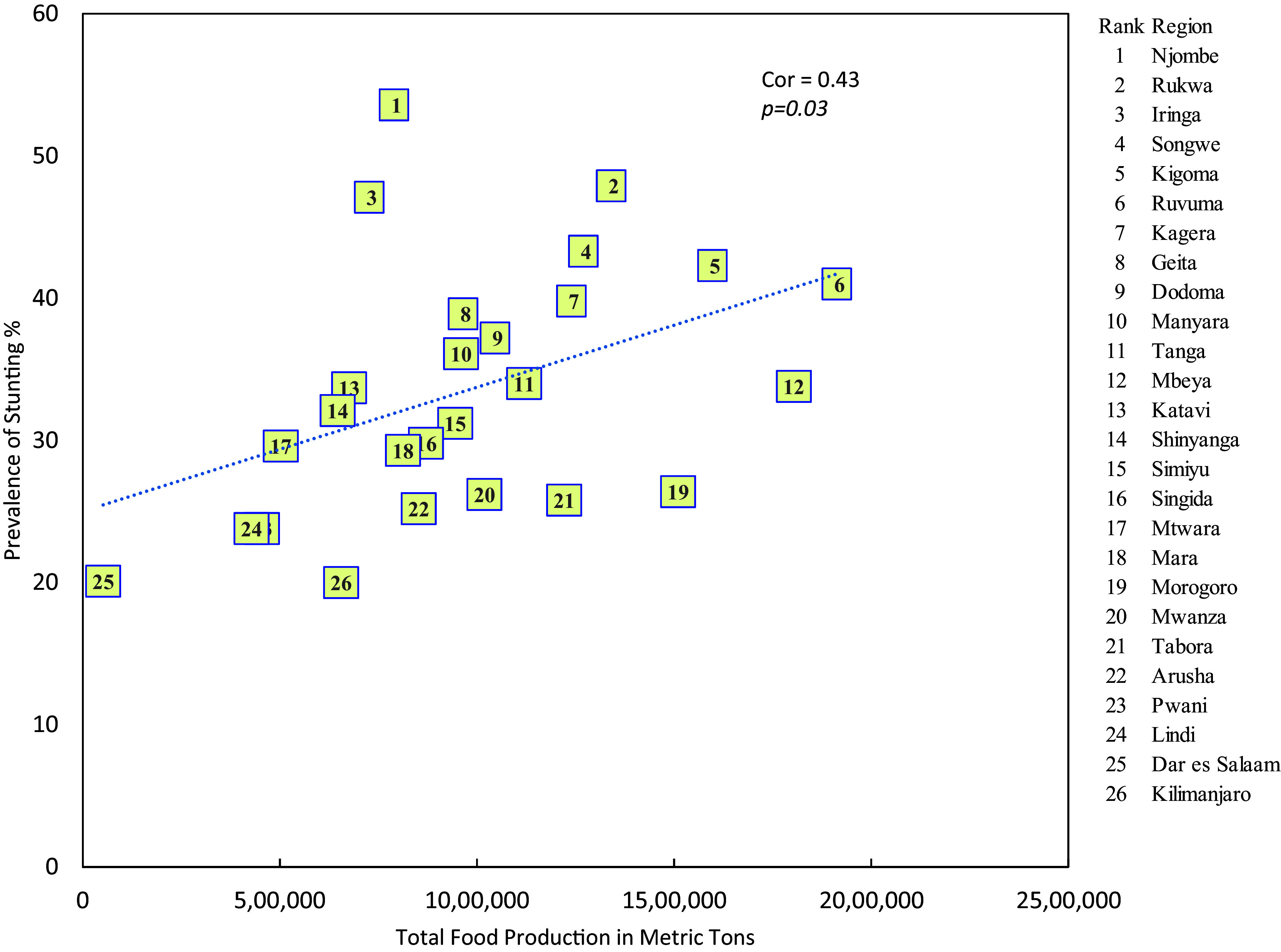
Prevalence of stunting and food production in mainland regions of Tanzania.

There is a significant positive linear relationship between the prevalence of stunting and the level of food production (r = 0.43, p = 0.03) i.e. the higher the level of food production the higher is the prevalence of stunting ( Figure 2).

### Stunting and average monthly household food consumption expenditure

**Figure 3.**
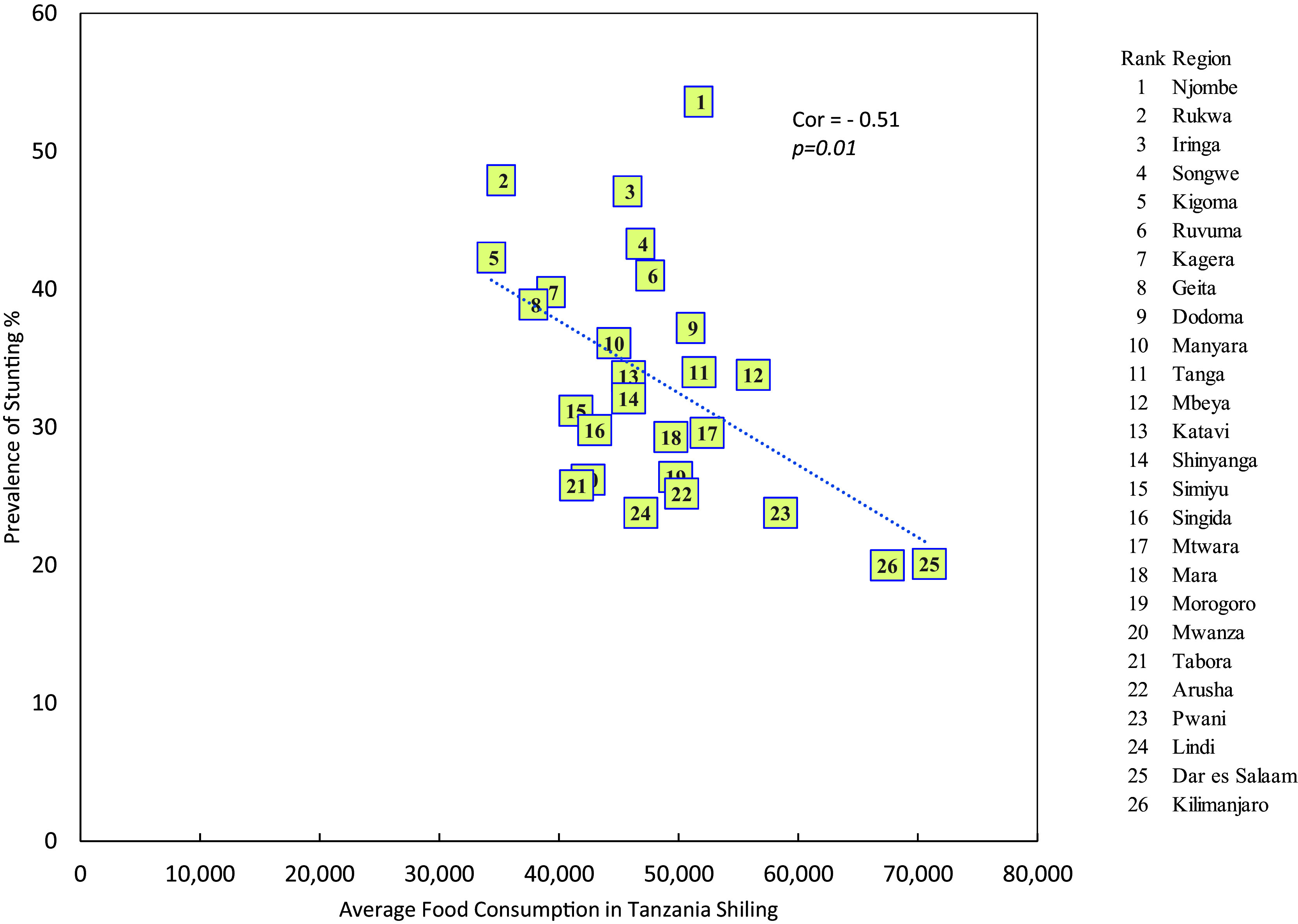
Prevalence of stunting by average monthly household food consumption expenditure in mainland regions of Tanzania.

The result indicated that there is a significant negative relationship between the prevalence of stunting and the average monthly food consumption expenditure levels per household per month (r = -0.51, p = 0.01) i.e. the higher the average monthly household food consumption expenditure, the higher is the prevalence of stunting (see Figure 3).

**Stunting and average monthly household non-food consumption expenditure**

**Figure 4.**
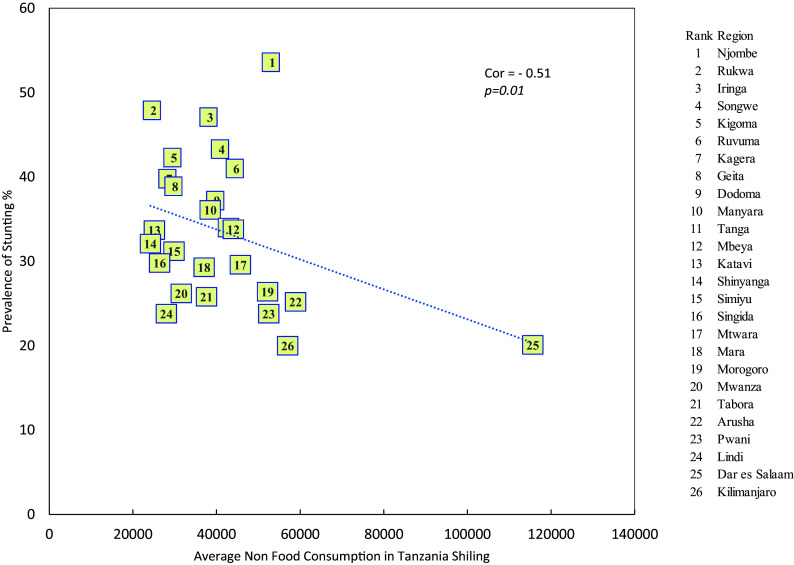
Stunting and average monthly household non-food consumption expenditure.

It was observed that there is a negative relationship between average monthly household non-food consumption expenditure and prevalence of stunting (r = -0.509, p = 0.01) i.e. as the average montly household non-food consumption expenditure increases the prevelence of stunting decreases (see Figure 4).

### Relationship Between Stunting and Basic Need Poverty

**Figure 5.**
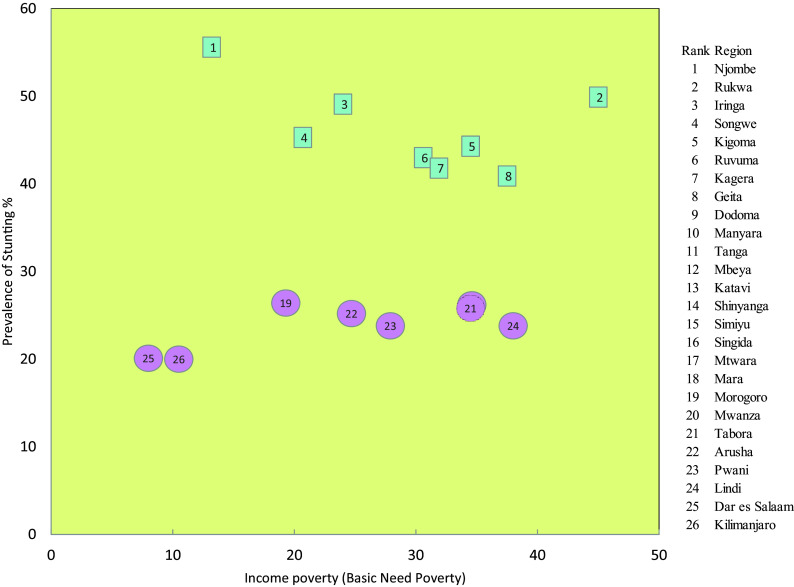
Stunting prevalence and basic need poverty in Tanzania Mainland (Blue squares 8 regions with high prevalence of stunting and 8 regions with lowest prevalence of stunting).

The relationship between stunting and basic needs poverty indicated that Njombe, which is the region with the highest level of stunting in the country (53%), is the region with the third lowest level of poverty (13.2%), next to Kilimanjaro (8%) and Dar es Salaam (10.5%). The prevalence of basic need poverty in Njombe, Iringa and Songwe, with stunting levels above 40%, is lower compared to other regions. However, the level of poverty and stunting in Rukwaa and Geita do not show notable differences. This trend is also observed for the regions with the lowest levels of stunting which are Kilimanjaro, Dar es Salama and Morogoro ( Figure 5).

## Discussion

Findings show that regions with the highest food production had a high prevalence of stunting. This result concurs with the study done in Bukombe, Tanzania, which found that children from peasants households producing plenty food were more likely to be stunted compared to other occupations. ^17^ This might be true because food-producing regions entail a great deal work, often done by mothers in their small scale farms, and hence little time is given for them to take care of and feed, their children. This indicates that food availability may not entail food consumption and related outcomes. This may mean that very low income households spend the majority of their time on the farm, producing and consuming one type of food and are thus severely affected by seasonal household food availability. Low income households may food available in the harvest season and food unavailabe in other seasons of the year while other occupations’ household may buy their food and hence have more food diversity. ^17^^,^^18^ However, these findings contrast other studies that have found households with food insecurity and small cultivation size, were more affected by stunting, compared to those who are food secure and those with large cultivated areas. ^9^^,^^19^

Our study found that some regions like Njombe, which are among the regions with the lowest levels of poverty, have higher levels of stunting. The findings are contrary to several studies done on the determinants of stunting in developing countries across the globe, which shows that poverty is positively associated with stunting. ^9^^–^^11^^,^^20^ The reason for this might be that mothers in regions like Njombe, are involved in several economic activities to earn their income. ^17^ Due to this, they may have limited time with their children. ^17^^,^^18^ In addition, labour laws in Tanzania, gives women only about three months’ maternity leave. ^21^ Therefore, due to mothers works in the farms or as employee in the govermenent or private institurions they do not have enough time to breastfed their children, take them to hospitals and feed them accordingly. ^17^^,^^18^

In addition, the results of the study show that regions with higher monthly household food and non-food consumption expenditure, had lower levels of stunting. This means that higher consumption means increased purchasing of a variety of foods, which reduces malnutrition. This finding concurs with a study done in Tanzania, which showed that families which were not dependent on farming their own food, bought a variety of food for their families, unlike those which produce their food. ^17^ Similarly, the family from farming family had lower dietary diversity (71%), compared to those from other occupation (55%). ^18^

## Conclusion and Recommendations

This study has found that the more the region produces food, the more likely it is to have higher levels of stunting. In addition, it has found that some regions with the lowest levels of poverty have higher levels of stunting. Regions with higher monthly household food and non-food consumption expenditure have higher levels of stunting. Therefore, this indicates that food production and a lower level of poverty, might not lead to nutrition outcomes. This study provides a picture of the relationship between food production; consumption, poverty and stunting in Tanzania. Therefore, it provides the foundation for further studies in this area. to understand contextual factors leading to high stunting in regions which have abountance of food. A comparative study can be done comparing regions with high food production with lower stunting level and those with higher level of food production performing poorly on stunting. Similarly, this information can enlighten policymakers on rethinking how to make agriculture nutrition-sensitive and education using of locally available food to attain nutrition outcomes especially diatary divirsity so that Tanzania can attain the Sustainable Development Goals (SDGs) goal of ending malnutrition in all its forms. ^22^

### Limitations of the study

This study faced a few limitations, one of them being that the study used secondary data of which some of the variables were missing, limiting our analysis. Furthermore, data were aggregated at the regional level rather than individual level, which limited the interpretation of factors associated with malnutrition. The food production included cereals and non-cereals. It did not include vegatables, fruits and animal products which are important for stunting reduction.

## Data availability

### Underlying data

Dryad: The Paradox of Food Production, Consumption, Poverty and Malnutrition in Tanzania

https://doi.org/10.5061/dryad.gxd2547n9

This project contains the following underlying data:

• Data_for_Regional_Stunting__Food_Production__Consumption_and_Poverty_Levels_-_Tanzania.xls

Data are available under the terms of the Creative Commons Zero “No rights reserved” data waiver (CC0 1.0 Public domain dedication).
