# Supplementary material for: The paradox of food production, expenditure, poverty and stunting  in Tanzania: an ecological study design
Source: F1000Res. 2026 Feb 26;11:32. Originally published 2022 Jan 12. [Version 3] doi: 10.12688/f1000research.74295.3 (PMC12993288; doi:10.12688/f1000research.74295.3)
Supplement: Peer review history

# Reviewer report: Reviewer response for version 3

**DOI:** 10.5256/f1000research.196830.r462961

**Published:** 2026-03-16

**Authors:** Zelda Anne Elum

**Affiliations:** 1 University of Port Harcourt, Choba, Rivers, Nigeria

The study result showing that the Tanzania regions which have high level of food production but associated with high stunting is an indication of lack of access to food. That is, the inability to purchase available food or consume other varieties of food. The implication is the need to improve access to food by improving household income and also the need to educate the people on food utilisation.

 Under the "Stunting and average monthly household food consumption expenditure" subheading, there is an incorrect statement as the negative correlation value should be interpreted as: "the higher the average monthly household food consumption expenditure, the lower is the prevalence of stunting". The paper contain other typos that need to be corrected.

 In addition, the negative correlation between the prevalence of stunting and household food consumption reinforces the importance of food accessibility or the affordability of food. As evidenced in the literature, peasant farmers often depend on their own production for household food consumption and they lack the means to afford other varieties of foods not produced. Conclusively, consistent food production addresses one dimension (availability) of food security. There is need for other dimensions (accessibility, utilisation and stability) to be improved.

Is the work clearly and accurately presented and does it cite the current literature?

Partly

If applicable, is the statistical analysis and its interpretation appropriate?

Yes

Are all the source data underlying the results available to ensure full reproducibility?

Yes

Is the study design appropriate and is the work technically sound?

Yes

Are the conclusions drawn adequately supported by the results?

Partly

Are sufficient details of methods and analysis provided to allow replication by others?

Partly

Reviewer Expertise:

Agricultural production economics, agricultural finance,  food security, gender studies, climate impacts on agriculture and adaptation.

I confirm that I have read this submission and believe that I have an appropriate level of expertise to confirm that it is of an acceptable scientific standard, however I have significant reservations, as outlined above.

# Reviewer report: Reviewer response for version 2

**DOI:** 10.5256/f1000research.196035.r456265

**Published:** 2026-02-12

**Authors:** Nahomi Imaeda

**Affiliations:** 1 Department of Nutrition, ​Shigakkan University, Aichi, Japan

Thank you for carefully addressing the previous comments. I appreciate that the revised manuscript now clearly specifies the regional names (e.g., Njombe), which substantially improves the transparency and interpretability of the findings. This clarification strengthens the ecological comparisons presented in the study.

 I would like to offer one additional suggestion to further enhance clarity in the abstract and to minimize potential ambiguity.

 In particular, the sentence:

 “It was further found that some regions which have higher levels of stunting such as Njombe, have the lowest level of basic need poverty.”

 may be somewhat difficult for readers to interpret unambiguously. The phrase “lowest level of basic need poverty” could be open to different interpretations, as it is not immediately clear whether this refers to poverty depth, poverty severity, or poverty rate.

 As “basic needs poverty” appears to represent the proportion of households unable to meet minimum consumption needs, it may improve clarity to explicitly refer to “poverty rates.” For example, the sentence could be revised as follows:

 “Furthermore, some regions with high stunting prevalence (e.g., Njombe) were found to have relatively low basic-needs poverty rates.”

 This wording more directly conveys the paradoxical pattern described in the title — namely, that regions with higher stunting do not necessarily correspond to regions with higher poverty rates — and would help readers more readily grasp the conceptual contrast highlighted in the study.

 Clarifying this expression would further strengthen the internal consistency between the title (“paradox”), the reported results, and their interpretation in the abstract.

 In addition, Figure 5 should ideally be self-explanatory without requiring reference to the main text. I would recommend adding a brief note in the figure legend clarifying the meaning of the blue squares and pink circles to improve readability.

**Overall, the manuscript has improved substantially, and with minor clarifications to wording and figure presentation, it will provide a clearer and more compelling account of the ecological paradox described.**

Is the work clearly and accurately presented and does it cite the current literature?

Yes

If applicable, is the statistical analysis and its interpretation appropriate?

Partly

Are all the source data underlying the results available to ensure full reproducibility?

Partly

Is the study design appropriate and is the work technically sound?

Yes

Are the conclusions drawn adequately supported by the results?

Partly

Are sufficient details of methods and analysis provided to allow replication by others?

Partly

Reviewer Expertise:

public health, dietary surveys, nutritional epidemiology

I confirm that I have read this submission and believe that I have an appropriate level of expertise to confirm that it is of an acceptable scientific standard.

# Response

**Published:** 2026-02-18

**Authors:** Novatus Tesha

**Affiliations:** Development Studies, Muhimbili University of Health and Allied Sciences, Dar es Salaam, Tanzania

We are thankful for the reviewer for taking part in reviewing our work. We are thankful for the comments provided, which continue to improve and make our work clearer. We agree with all the comments raised and have addressed them as shown below.

**Reviewer comment 1**

 Thank you for carefully addressing the previous comments. I appreciate that the revised manuscript now clearly specifies the regional names (e.g., Njombe), which substantially improves the transparency and interpretability of the findings. This clarification strengthens the ecological comparisons presented in the study.

 I would like to offer one additional suggestion to further enhance clarity in the abstract and to minimize potential ambiguity.

 In particular, the sentence:

 “It was further found that some regions which have higher levels of stunting such as Njombe, have the lowest level of basic need poverty.”

 may be somewhat difficult for readers to interpret unambiguously. The phrase “lowest level of basic need poverty” could be open to different interpretations, as it is not immediately clear whether this refers to poverty depth, poverty severity, or poverty rate.

 As “basic needs poverty” appears to represent the proportion of households unable to meet minimum consumption needs, it may improve clarity to explicitly refer to “poverty rates.” For example, the sentence could be revised as follows:

 “Furthermore, some regions with high stunting prevalence (e.g., Njombe) were found to have relatively low basic-needs poverty rates.”

 This wording more directly conveys the paradoxical pattern described in the title — namely, that regions with higher stunting do not necessarily correspond to regions with higher poverty rates — and would help readers more readily grasp the conceptual contrast highlighted in the study.

 Clarifying this expression would further strengthen the internal consistency between the title (“paradox”), the reported results, and their interpretation in the abstract.

**Response**

 We are very thankful to the reviewer for these comments. We agree with the reviewer, and we have made changes to the abstract to include these suggestions.

**Reviewer comment 2**

 In addition, Figure 5 should ideally be self-explanatory without requiring reference to the main text. I would recommend adding a brief note in the figure legend clarifying the meaning of the blue squares and pink circles to improve readability.

 Thank you. We agree with the reviewer and have added a note to Figure 5. The figure legend is now " Figure 5. Stunting prevalence and basic need poverty in Tanzania Mainland (Blue squares: 8 regions with high prevalence of stunting and 8 regions with the lowest prevalence of stunting)

**Reviewer comment**

 Overall, the manuscript has improved substantially, and with minor clarifications to wording and figure presentation, it will provide a clearer and more compelling account of the ecological paradox described.

**Response**

 Thank you

# Reviewer report: Reviewer response for version 1

**DOI:** 10.5256/f1000research.78033.r144313

**Published:** 2022-08-10

**Authors:** Nahomi Imaeda

**Affiliations:** 1 Department of Nutrition, ​Shigakkan University, Aichi, Japan

Despite the need for epidemiological studies targeting developing countries, there are still few publications. In this context, an ecological study design that allows low-cost analysis would be beneficial. I read this paper with great interest.

 Ecological studies cannot prove causality, but the author should choose appropriate parameters to clarify the relationship with the prevalence of stunting. The author describes it as a paradox that the food production value is positively correlated with stunting, but the indicators observed are not appropriate. The author should observe in more detail what food groups are produced. For example, the production of coffee beans would not be directly related to an increase in nutrient intake.

 Furthermore, for mothers in Region Njombe, the author should show statistical data on longer hours of work in comparison with other areas.

 In Figures 1-3:

- The graphs presenting the 26 regions should show approximate equations. In addition, for those regions that had large residuals from the equation, the reasons should be discussed.
- The figures should be drawn squares with numbers like [1], [2] - [26], indicating the name of the region, rather than a square black box, so that the stunting situation and economic situation in Tanzania can be understood.

Since most of the readers have unknown area names in Tanzania, I suppose that as possible as simple maps should be shown with transport infrastructure (road network or ocean ports). In this peer review, I was able to get some Tanzania information by the administrative regions of Tanzania map (referred Tanzania National Nutrition Survey 2018.pdf, page 7, Figure 1), or the Tanzania transport map (website: https://www.tanzaniainvest.com/transport ). The figures of present paper did not provide enough information about regions in Tanzania.

 In Figures 4 and 5, only 8 regions were observed, so the explanation is not sufficient.

 The keyword “basic needs poverty” should be changed to “basic human needs” and “poverty”?  or “basic needs poverty line”?  And according to the PubMed Mesh, the keyword " stunting" is included in Growth Disorders. I recommend you refer to the PubMed Mesh to increase the number of web-search hits.

Is the work clearly and accurately presented and does it cite the current literature?

Yes

If applicable, is the statistical analysis and its interpretation appropriate?

Partly

Are all the source data underlying the results available to ensure full reproducibility?

Partly

Is the study design appropriate and is the work technically sound?

Yes

Are the conclusions drawn adequately supported by the results?

Partly

Are sufficient details of methods and analysis provided to allow replication by others?

Partly

Reviewer Expertise:

public health, dietary surveys, nutritional epidemiology

I confirm that I have read this submission and believe that I have an appropriate level of expertise to confirm that it is of an acceptable scientific standard, however I have significant reservations, as outlined above.

# Response

**Published:** 2026-01-31

**Authors:** Novatus Tesha

**Affiliations:** Development Studies, Muhimbili University of Health and Allied Sciences, Dar es Salaam, Tanzania

We are very thankful to the reviewer for reviewing our work. The reviewers' comments and inputs have been crucial in improving our work. Below, we provide point-by-point responses to reviewers' comments, which we have all agreed on and worked on.****

 1. Despite the need for epidemiological studies targeting developing countries, there are still few publications. In this context, an ecological study design that allows low-cost analysis would be beneficial. I read this paper with great interest.

 Response 

 Thank you

 2. Ecological studies cannot prove causality, but the author should choose appropriate parameters to clarify the relationship with the prevalence of stunting. The author describes it as a paradox that the food production value is positively correlated with stunting, but the indicators observed are not appropriate. The author should observe in more detail what food groups are produced. For example, the production of coffee beans would not be directly related to an increase in nutrient intake.

 Response 

 Thank you very much, the data we have used are for food that are non-commercial. This includes cereals and non-cereals. The non-cereals include beans, pigeon peas, cowpeas, groundnuts, greengram, bambaranus, cassava, banana, sweet potatoes and round potatoes. Have added this explanation in the method section. Page 5

 3. The graphs presenting the 26 regions should show approximate equations. In addition, for those regions that had large residuals from the equation, the reasons should be discussed

 Thank you, all figures have been changed. Pages 7-10

 4. The figures should be drawn squares with numbers like [1], [2] - [26], indicating the name of the region, rather than a square black box, so that the stunting situation and economic situation in Tanzania can be understood.

 Response 

 Thank you very much, this has been applied to all figures. Pages 7-10

 5 Since most of the readers have unknown area names in Tanzania, I suppose that as possible as simple maps should be shown with transport infrastructure (road network or ocean ports). In this peer review, I was able to get some Tanzania information by the administrative regions of Tanzania map (referred Tanzania National Nutrition Survey 2018.pdf, page 7, Figure 1), or the Tanzania transport map (website: https://www.tanzaniainvest.com/transport ). The figures of present paper did not provide enough information about regions in Tanzania.

 Response 

 Thank you, we have added Figure 1 from Tanzania National Nutrition Survey 2018.pdf, page 7, Figure 1.

  Page 4

 6. In Figures 4 and 5, only 8 regions were observed, so the explanation is not sufficient.

 Response 

 Thank you, the figures now include all regions of Tanzania Page 10

 7. The keyword “basic needs poverty” should be changed to “basic human needs” and “poverty”?  or “basic needs poverty line”?  And according to the PubMed Mesh, the keyword " stunting" is included in Growth Disorders. I recommend you refer to the PubMed Mesh to increase the number of web-search hits.

 Response 

 Thank you. We have used these terms based on the reports from which it was obtained for easy of reference.

# Reviewer report: Reviewer response for version 1

**DOI:** 10.5256/f1000research.78033.r123223

**Published:** 2022-03-14

**Authors:** Joyce Kinabo

**Affiliations:** 1 Department of Food Technology Nutrition and Consumer Sciences, College of Agriculture, Sokoine University of Agriculture, Morogoro, Tanzania

**Commendation**:

 I would like to commend the authors for coming up with this paper in which they have attempted to provide some answers about the paradox relationship between food production, stunting, expenditure, and poverty in Tanzania. They have correctly pointed out that high food producing regions are also leading in the prevalence of stunting.

**Suggestion**: change the title to read “the paradox of food production, expenditure, poverty, and stunting in Tanzania….” To reflect what is described in the paper.

**Methodology:**

- Sampling procedure for the Household Budget Survey and Tanzania National Nutrition Survey has been described but not for the agriculture statistics. The authors should explain how the data on agriculture production is collected. It would also be useful to indicate when these various surveys were carried out (date and month). This information has implications on the relationships of the variables and therefore interpretation of the results.
- Provide the definition of average consumption. The household budget survey presents proportion of food and non-food expenditures, it would be more logical to look into these categories and assess how they relate to stunting instead of just looking at the average expenditure and expenditure on food only. In addition, what are the kinds of foods that are accessed by households? Do they add to the diversity of nutritious foods?

**Results:**

- Add lines of fit on the correlation graphs (scatter plots) to assist the reader to get a clear picture of the relationship between the variables.
- Stunting and average monthly household consumption expenditure (AMHCE): the authors have stated that there was a significant negative relationship between stunting and AMHCE; but explained that as AMCHE increased, stunting also increased. Is this the meaning of a negative relationship? This area needs clarification.
- Stunting and AMHFCE: authors observed a negative relationship, but did not elaborate further on this relationship the way they have done for other relationships. There is a need for consistency.
- Stunting and basic need poverty: what is the basis of selecting only eight regions. It is better to present data for all regions to give the reader a full perspective of the data you have analysed and single out the eight regions in the description of the results. In the current analysis, the Njombe region should appear in both figures 4 and 5. However, it would also be interesting to identify regions that appear in both figures 4&5.
- It would also be more informative to present data on the relationship between stunting and monthly food and non food expenditures to show how the two types of expenditure contribute to stunting. Nevertheless, this is not a very strong indicator since it does not tell us what types of foods are purchased and consumed by children.

**Discussion**

- “This indicates that food security may not entail nutrition security” This is a statement in the discussion, but not explained fully. Authors should be cautious when using these terminologies. In this particular study, they assessed the relationship between food production or availability and stunting and not food security, which has four pillars. It would be better to say that food availability may not entail food consumption and related outcomes; rather than using words such as food and nutrition security, which were not assessed in this study.
- Page 7 para 3 is more speculative and not supported by the available data. It should be revised to provide more information about expenditure and what kinds of foods are purchased and actually fed to children. It would also be more logical to consider other variables such as minimum acceptable diet (MAD) for children and household dietary diversity score (HDDS).

**Conclusion**:

- The authors have not been able to explain the paradox. The conclusion is the same as introduction. It was expected to get an explanation about the paradox and how that can be corrected.

Is the work clearly and accurately presented and does it cite the current literature?

Partly

If applicable, is the statistical analysis and its interpretation appropriate?

Partly

Are all the source data underlying the results available to ensure full reproducibility?

Yes

Is the study design appropriate and is the work technically sound?

Yes

Are the conclusions drawn adequately supported by the results?

Partly

Are sufficient details of methods and analysis provided to allow replication by others?

Partly

Reviewer Expertise:

NA

I confirm that I have read this submission and believe that I have an appropriate level of expertise to confirm that it is of an acceptable scientific standard, however I have significant reservations, as outlined above.

# Response

**Published:** 2026-01-31

**Authors:** Novatus Tesha

**Affiliations:** Development Studies, Muhimbili University of Health and Allied Sciences, Dar es Salaam, Tanzania

We are very thankful to the reviewer who reviewed our work. We agree with all comments and have provided a point-by-point response detailing how we have addressed them. These comments have improved our work.

**Commendation**

 1. I would like to commend the authors for coming up with this paper, in which they have attempted to provide some answers about the paradox relationship between food production, stunting, expenditure, and poverty in Tanzania. They have correctly pointed out that high food-producing regions are also leading in the prevalence of stunting

 Response 

 Thank you

**Tittle**

 2. Change the title to read “the paradox of food production, expenditure, poverty, and stunting in Tanzania….” To reflect what is described in the paper

 Response 

 Thank you, we agree with the reviewer, the title has been revised as suggested.

 N/A

**Methodology**

 3. Sampling procedure for the Household Budget Survey and Tanzania National Nutrition Survey has been described but not for the agriculture statistics. The authors should explain how the data on agriculture production is collected. It would also be useful to indicate when these various surveys were carried out (date and month). This information has implications on the relationships of the variables and therefore interpretation of the results.

 Response

 Thank you, we agree with the reviewer. The survey dates have been added: HBS 1st December 2017 to 30th November 2018 and TNNS 17th of November 2018. Regarding the agriculture statistics, it is a synthesis report which collects reports from 10 different tools collected from all districts of the country. The report does not give details on the dates and how each tools sampled its population. Pages

  4 & 5

 4. Provide the definition of average consumption. The household budget survey presents proportion of food and non-food expenditures, it would be more logical to look into these categories and assess how they relate to stunting instead of just looking at the average expenditure and expenditure on food only. In addition, what are the kinds of foods that are accessed by households? Do they add to the diversity of nutritious foods?

 Response

 Thank you, we agree with the reviewer. We have added the average household expenditure on non-food and removed the average consumption variable. In case of food diversity, we have added an explanation on the details on the food that were included in the food production variable. Pages

 5, 7 - 9

**Findings**

 5. Add lines of fit on the correlation graphs (scatter plots) to assist the reader to get a clear picture of the relationship between the variables

 Response 

 Thank you, this have been added throughout all figures.  Pages 8-10

 6. Stunting and average monthly household consumption expenditure (AMHCE): the authors have stated that there was a significant negative relationship between stunting and AMHCE; but explained that as AMCHE increased, stunting also increased. Is this the meaning of a negative relationship? This area needs clarification.

 Response

 Thank you very much there was a typo. We agree with the reviewer , however, we have removed this variable so that food and non-food household expenditure can be reported. Pages 7-9

 7. Stunting and AMHFCE: authors observed a negative relationship, but did not elaborate further on this relationship the way they have done for other relationships. There is a need for consistence

 Response

 Thank you very much we have added an elaboration of the relationship. Page 8

 8. Stunting and basic need poverty: what is the basis of selecting only eight regions. It is better to present data for all regions to give the reader a full perspective of the data you have analysed and single out the eight regions in the description of the results. In the current analysis, the Njombe region should appear in both figures 4 and 5. However, it would also be interesting to identify regions that appear in both figures 4&5.

 Response 

 Thank you, all 25 regions have now been presented in the figures. Pages 9-10

 9 It would also be more informative to present data on the relationship between stunting and monthly food and non food expenditures to show how the two types of expenditure contribute to stunting. Nevertheless, this is not a very strong indicator since it does not tell us what types of foods are purchased and consumed by children

 Response

 Thank you. We agree with the reviewer. We have added non-food expenditure in our analysis. Pages 7-9

**Discussions**

 10 “This indicates that food security may not entail nutrition security” This is a statement in the discussion, but not explained fully. Authors should be cautious when using these terminologies. In this particular study, they assessed the relationship between food production or availability and stunting and not food security, which has four pillars. It would be better to say that food availability may not entail food consumption and related outcomes; rather than using words such as food and nutrition security, which were not assessed in this study

 Response 

 Thank you. We agree with the reviewer and edited the section to food availability may not entail food consumption and related outcomes. Page 10 and throughout the discussion section

 11 Page 7 para 3 is more speculative and not supported by the available data. It should be revised to provide more information about expenditure and what kinds of foods are purchased and actually fed to children. It would also be more logical to consider other variables such as minimum acceptable diet (MAD) for children and household dietary diversity score (HDDS)

 Response 

 Thank you very much, we have added the findings on dietary divisity which found children from farming household performed lower on diatary diversity compared to other occupation. Page 11

**Conclusion**

 12 The authors have not been able to explain the paradox. The conclusion is the same as introduction. It was expected to get an explanation about the paradox and how that can be corrected

 Response 

 Thank you, we agree with the reviewer. We have edited the conclusion. We have added research specific recommendation and the policy recommendation for understanding further the the contextual factors for the paradox and solutions to the paradox. Page  11
